# An Overview of the Relationship Between Occupational Manganese Exposure and Parkinsonism

**DOI:** 10.7759/cureus.32161

**Published:** 2022-12-03

**Authors:** Elham Dirandeh, Ali Palizgir, Negin Kassiri

**Affiliations:** 1 Department of Neurology, Shariati Hospital, Tehran University of Medical Sciences, Tehran, IRN; 2 Department of Occupational Medicine, School of Medicine, Iran University of Medical Sciences, Tehran, IRN; 3 Occupational Health Research Center, Department of Occupational Medicine, School of Medicine, Iran University of Medical Sciences, Tehran, IRN

**Keywords:** occupational disease, occupational health, neurotoxic, metal, manganese, parkinsonism

## Abstract

Manganese (Mn) is an essential element used in many industries, such as welding, foundries, the production of metal alloys, especially stainless steel, and the production of dry batteries, pesticides, paints, and explosives. Individuals are exposed to Mn through inhalation of fumes, dermal absorption, and ingestion. This metal is an essential trace element required for normal growth, development, and cellular homeostasis. It has also toxic effects on the central nervous system and can cause Parkinsonism symptoms in exposed patients. Studies on human and animal models reveal that neurons of the globus pallidus, the cerebellum, pons, red nucleus, the thalamus, cortex, and the anterior horn of the spinal cord could be affected by Mn toxicity. Although the diagnosis of manganese-induced Parkinsonism is primarily clinical, there are some supporting features on brain MRI images that may be helpful to objectively distinguish it. This study was designed to review the ways of exposure to Mn, clinical symptoms in case of exposure, and discover the relationship between exposure to Mn and Parkinsonism in the working population.

## Introduction and background

Trace metals, such as manganese (Mn), zinc (Zn), copper (Cu), and iron (Fe), are essential elements and have low concentrations in the human body. They play a role of cofactors in a wide range of physiological processes and are involved in cellular homeostasis [1]. Mn is an essential, ubiquitous trace element required for normal growth, development, and cellular homeostasis [1-5]. Individuals are exposed to Mn through inhalation of fumes, dermal absorption, and ingestion [1,4,6]. Whole grains, nuts, seeds, beans, pineapple, tea, and legumes are food sources that contain Mn [1,3,5,7,8]. This black, brittle metal with an atomic number of 25 known as ferromanganese (a combination of iron and manganese) is widely used in the production of metal alloys, especially stainless steel [2,7].

Most welding materials used in the mild steel welding process contain less than 6% of Mn [9]. Also, this metal is used in the production of dry batteries, as an oxidizing agent in the metal industry, in the production of pesticides, paints, and explosives (the combustible part of matches), and organic Mn is used in petrochemical industries and refineries as an anti-explosion and anti-combustion agent [2,8]. At excessive levels, Mn is toxic to the central nervous system (CNS) [1]. An individual's symptoms usually start with headache and fatigue, followed by irritability, increased libido, and psychosis. If exposure continues, signs of Parkinsonism will appear [10]. The motor impairments include bradykinesia, hypertonia with cogwheel rigidity, stooped posture, cock-gait, rapid postural tremor, decreased arm swing, and postural instability. Long-term inhalation of Mn fumes may lead to Mn-induced occupational Parkinsonism.

More than 90% of the Mn-containing aerosols have a size smaller than 10 microns and can be deposited in the lungs. The worldwide prevalence of occupationally mediated Mn Parkinsonism is poorly understood. China’s rate is estimated to be about 0.5%-2% in silico- and ferromanganese factory workers. Also, in American studies, in states with high industrial Mn emissions, an increase in the incidence of neurological symptoms caused by Mn and mortality related to it was shown [5].

Considering that exposure to Mn is present in different industrial job categories and that most of the exposed workers are young, and also considering that Mn toxicity can cause symptoms and signs of Parkinsonism, it is important to research the correlation between Mn toxicity and probable Parkinsonism. Studies are not enough in this field. Moreover, the direct and indirect costs of this disease impose a heavy economic burden on governments and countries. Hence, we decided to discuss the frequency of Parkinsonism in different occupational populations exposed to Mn and their correlation.

## Review

Search strategy

In this review, we gathered human epidemiologic and case-control evidence for individual environmental agents associated with Parkinsonism. Articles from peer-reviewed scientific journals were selected from databases including PubMed, Google Scholar, Scopus, and ProQuest; search phrases such as “Mn exposure”, “Parkinsonism”, and “occupational diseases”, in titles or abstracts, were used for studies until August 2022. After completing the research process, duplicate and similar articles were removed and the related ones were separated and reviewed.

Mn, a trace element

The daily consumption of food sources containing Mn regulates several basic physiological processes such as blood sugar regulation, reproduction, bone hemostasis, metabolism of lipids, proteins, and carbohydrates, and immune response [1,3,5]. Mn is a cofactor for enzymes such as pyruvate decarboxylase, glutamine synthetase, protein serine/threonine phosphatase I, Mn superoxide dismutase (Mn-SOD), and arginase, which are involved in neurotransmitter synthesis, metabolism, and neuronal function [3,5]. It is usually excreted through bile, and accordingly, biological monitoring for this trace metal is hard to achieve. The half-life is about 30 hours in the body, and it can be detected in the blood, urine, nails, and hair of exposed people, but the amount of Mn that is detected is not related to the severity of toxicity symptoms.

Mn toxicity

More than one million workers around the world are exposed to Mn fumes in various ways. Previous studies have suggested that about 62%-72% of American welders are exposed to levels above the limit set by the American Conference of Governmental Industrial Hygienists (ACGIH) [11-13]. In a study on foundry workers, blood and environmental Mn levels were measured over 16 months. It was found that foundry workers working in poorly ventilated environments had significant blood Mn levels (2.5-5 μg/l above the reference limit) despite low exposure levels (environmental Mn levels, 0.002-0.064 mg/m^3^). The blood level of Mn is a good indicator of recent exposure (about one to two weeks before testing) [14].

In acute exposure to Mn toxicity, it can cause symptoms such as skin burns, headaches, fatigue, a metallic taste in the mouth, shortness of breath, and chest pain; in severe forms, chemical pneumonia and acute damage to the liver and kidneys occur. During chronic exposure, neurological symptoms, increased sweating, saliva production, involvement of the vasomotor system, and increased risk of respiratory infections have been reported [6].

Mn exposure and Parkinsonism

Mn is known as a neurotoxic substance that can lead to a severe and atypical form of Parkinsonian syndrome. Mn leaves its neurotoxic effects by disrupting mitochondrial oxidative metabolism, disabling cellular antioxidant defense, and inducing dopaminergic toxicity [15]. In 1837, during the examination of workers who were exposed to Mn dioxide in an Mn ore-crushing factory, Couper described Mn-induced Parkinsonism for the first time [3,5,8]. The toxicity of this metal is characterized by motor and sensory disturbances, cognitive deficits, and neuropsychiatric symptoms [1,16]. Several studies have shown that exposure to Mn fumes, even at levels lower than the Occupational Safety and Health Administration (OSHA) standard (5 mg/m^3^), is associated with the mentioned symptoms [17-19]. Studies on human and animal models reveal that neurons of the globus pallidus are most sensitive to Mn-induced degeneration. Other brain regions, including the cerebellum, pons, red nucleus, thalamus, cortex, and anterior horn of the spinal cord, could be affected by Mn toxicity [1,3,8,16,19-21].

A significant amount of evidence clearly indicates that Mn intoxication, either of environmental (occupational or drug-induced) or genetic origin, results in atypical Parkinsonism associated mainly with dystonia, rigidity, fine motor control deficits, cock-walk gait, and speech disturbances [19,22]. The mechanism by which Mn leads to Parkinsonism is poorly understood. Mn primarily targets the globus pallidus internus, the striatum, and the substantia nigra pars reticulata. In rare cases, it may affect the caudate and putamen, whereas the substantia nigra pars compacta, the primary target of idiopathic Parkinson’s disease, is spared [9,22].

Although the diagnosis of Mn-induced Parkinsonism is primarily clinical, there are some supporting features that may be helpful to objectively distinguish it. Magnetic resonance imaging (MRI) is a useful and low-risk tool that is used for detecting Mn deposition in the brain regions and other organs. On brain MRI, T1-weighted images show bilateral hyperintensity of the globus pallidus [7,9,16,20,21]. Caudate, putamen, and substantia nigra could be involved. Yet a clear fundamental indicator of manganism has not been identified in neurobehavioral studies [9].

The relationship between exposure to Mn and neurological symptoms is contradictory and the frequency of Parkinsonism has been reported to vary in different occupational populations exposed to Mn.

Refusal Studies

Several studies do not confirm the relationship between occupational exposure to Mn (such as welding, casting, etc.) and Parkinsonism. In a study by Goldman and colleagues in 2005, the frequency of Parkinsonism in different occupations was investigated. They examined the results of 2249 patients and concluded that the frequency of occupations such as health service workers, teachers, and farmers was higher in patients with Parkinsonism (occupations with no Mn exposure) [23]. In a cohort study conducted to find the relationship between welding and movement and basal ganglia disorders, all welders and cutters between 1960 and 1970 were examined for the occurrence of movement and basal ganglia disorders between 1964 and 2003 and compared with a control group that was age and geographically matched. Finally, it became clear that the rate of movement and basal ganglia disorders in the population of welders and cutters was similar to that in the general population (adjusted rate ratio, or aRR,   0.91; 95% confidence interval, or CI, 0.81-1.01). The incidence of Parkinson’s disease in shipyard welders exposed to high levels of welding fumes was also the same compared to the general population (aRR   0.95; 95% CI 0.70-1.28). Hence, this study did not confirm the relationship between welding and Parkinson’s disease or any movement or basal ganglia disorders [24]. A previous cohort study of 27,839 Danish metal workers from 1977 to 2002 found no increased hospitalizations for Parkinson’s disease or other neurological conditions. The standardized hospitalization ratio and 95% CI for Parkinson’s disease for men in steel-manufacturing companies, welding departments, and welders were 1.0 (CI 0.7-1.5), 0.9 (CI 0.7-1.2), and 0.9 (CI 0.4-1.5), respectively [25]. In another cohort study conducted to assess the risk of Parkinson’s disease and other neurodegenerative diseases, 5867 Danish welding workers were compared with 1735 Danish non-welding workers. They concluded that welders were not at increased risk for Parkinson’s disease [26]. In a 2012 meta-analysis, the association between Mn exposure and Parkinsonism was evaluated. The relative risk was found to be 0.86% and the odds ratio (OR) was found to be 0.76%. Therefore, it was concluded that welding, exposure to Mn, and Parkinson’s disease were not related, and the expected association was probably due to the presence of confounding factors such as smoking, the healthy worker effect, or the effect of exposure dose (Mn is harmful in medium to high doses and beneficial in low doses) [27].

Confirmatory Studies

There are several studies confirming the association between exposure to Mn and Parkinsonism. In a 2005 cross-sectional study by Racette et al., the prevalence of Parkinsonism in welders was compared to that in the general population. The prevalence of Parkinsonism among male workers aged 40 to 69 years was estimated to be 977 to 1336 per 100,000. After adjusting for age, it was found that the prevalence of Parkinsonism among welders was higher than that of the general population [28]. In another study done in 2012 to identify the prevalence of Parkinsonism, 811 shipyard and construction welders were evaluated. The control group consisted of 59 non-welder workers and 118 untreated patients diagnosed with idiopathic Parkinson’s disease. Individuals were evaluated by a neurologist who was an expert in movement disorders, and the Unified Parkinson’s Disease Rating Scale, motor subsection 3 (UPDRS-3), score was determined. Patients with a score higher than 15 were considered Parkinsonism cases and those with a score less than 6 were considered normal cases. The prevalence of Parkinsonism was estimated at 15.6% in the group of welders compared to 0% in the reference group. In the group of welders, a U-shaped dose-response relationship was reported between exposure years and Parkinsonism. UPDRS-3 scores were mostly similar between the group of welders and patients with newly diagnosed idiopathic Parkinson’s disease, except for resting tremor and asymmetry, which were more commonly reported in patients with Parkinson’s disease [29].

In a 2015 study by Andruska and Racette, the prevalence of Parkinson’s disease was reported to be about 2% in the general population over 65 years of age. This study, by examining the phenotype of 716 welding workers who were exposed to Mn, compared to the non-welder control group, estimated the prevalence of Parkinsonism in welders’ group to be about 15.6% [30]. Another study on 886 welders announced that the prevalence of Parkinsonism using the UPDRS-3 method was 15.2% [31]. A cohort study of 886 American welders, over a follow-up period of nearly 10 years, showed that with increasing cumulative doses of Mn, the progression of Parkinsonism included the progression of bradykinesia, rigidity, speech defects, and facial expression [32]. In order to discover the relationship between exposure to Mn, Parkinsonism, and quality of life, a study was conducted that included the population of Mn miners in South Africa. In this study, information on 418 Mn miners was collected from 2010 to 2014. Parkinsonism was considered the primary outcome, defined by a UPDRS-3 score greater than 15. The health status of miners or the quality of life was also proposed as the second result, which was evaluated with the 39-item Parkinson’s Disease Questionnaire (PDQ-39). The prevalence of Parkinsonism was reported to be 29.4%. The PDQ-39 scores were higher in employees with Parkinsonism. No evidence was found for a monotonic pattern of a dose-response relationship between cumulative Mn exposure and Parkinsonism and quality of life. Age over 40 years (with OR 2.11, 95% CI 1.18-3.78) was a strong predictor for Parkinsonism. Parkinsonism (p = 0.004) and age (p = 0.031) were strong predictors of a decreased quality of life [33]. In an Asian cross-sectional study including 83 male foundry workers, the prevalence of Parkinsonism through clinical examination and transcranial sonography findings was reported at 42.2%. Parkinsonism according to lentiform nucleus hyperechogenicity had an association with smoking history (OR 26.63, 95% CI 2.38-178.71) and work experience (OR 7.18, 95% CI 0.84-61.32) [34].

It can be seen that studies had different opinions about this relationship. While a group of studies rejected this association and considered the prevalence of Parkinsonism in occupations exposed to Mn to be similar to that in the general population, another group of studies supported such a relationship and reported the prevalence of Parkinsonism caused by Mn to be between 15% and 42%. Considering that most studies with high sample sizes confirmed this association, it is suggested that longitudinal studies with high sample sizes and in different occupational populations should be conducted in the future.

## Conclusions

Mn is an essential trace element required for normal growth and development in humans, but it can also have a neurotoxic effect on the CNS at excessive levels. Although the diagnosis of manganese-induced Parkinsonism is primarily clinical, brain MRI images may be helpful to objectively distinguish it; T1-weighted images show bilateral hyperintensities of the globus pallidus, caudate, putamen, and substantia nigra. Keeping in mind the importance of disease incidence in the working population, which is often at a young age, control measures, such as removing occupational hazards as much as possible, management measures, technical engineering, use of proper ventilation, observance of the work and rest cycle, employee training, and use of appropriate personal protective equipment, are recommended.

## References

[1] Mezzaroba L, Alfieri DF, Colado Simão AN, Vissoci Reiche EM (2019). The role of zinc, copper, manganese and iron in neurodegenerative diseases. Neurotoxicology.

[2] Jankovic J (2005). Searching for a relationship between manganese and welding and Parkinson's disease. Neurology.

[3] Tuschl K, Mills PB, Clayton PT (2013). Manganese and the brain. Int Rev Neurobiol.

[4] Racette BA, Nelson G, Dlamini WW (2021). Severity of parkinsonism associated with environmental manganese exposure. Environ Health.

[5] Kwakye GF, Paoliello MM, Mukhopadhyay S, Bowman AB, Aschner M (2015). Manganese-induced parkinsonism and Parkinson's disease: shared and distinguishable features. Int J Environ Res Public Health.

[6] Segura Aguilar J, Kostrzewa RM (2004). Neurotoxins and neurotoxic species implicated in neurodegeneration. Neurotox Res.

[7] McKnight S, Hack N (2020). Toxin-Induced parkinsonism. Neurol Clin.

[8] Parmalee NL, Aschner M (2016). Manganese and aging. Neurotoxicology.

[9] Furbee B (2011). Welding and parkinsonism. Neurol Clin.

[10] Guilarte TR (2010). Manganese and Parkinson's disease: a critical review and new findings. Environ Health Perspect.

[11] Castleman BI, Ziem GE (1994). American conference of governmental industrial hygienists: low threshold of credibility. Am J Ind Med.

[12] Korczynski RE (2000). Occupational health concerns in the welding industry. Appl Occup Environ Hyg.

[13] Susi P, Goldberg M, Barnes P, Stafford E (2000). The use of a Task-Based Exposure Assessment Model (T-BEAM) for assessment of metal fume exposures during welding and thermal cutting. Appl Occup Environ Hyg.

[14] Lander F, Kristiansen J, Lauritsen JM (1999). Manganese exposure in foundry furnacemen and scrap recycling workers. Int Arch Occup Environ Health.

[15] HaMai D, Bondy SC (2004). Oxidative basis of manganese neurotoxicity. Ann N Y Acad Sci.

[16] Criswell SR, Nielsen SS, Warden MN (2019). MRI signal intensity and parkinsonism in manganese-exposed workers. J Occup Environ Med.

[17] Bouchard M, Mergler D, Baldwin M, Panisset M, Bowler R, Roels HA (2007). Neurobehavioral functioning after cessation of manganese exposure: a follow-up after 14 years. Am J Ind Med.

[18] Laohaudomchok W, Lin X, Herrick RF (2011). Neuropsychological effects of low-level manganese exposure in welders. Neurotoxicology.

[19] Guilarte TR, Gonzales KK (2015). Manganese-induced Parkinsonism is not idiopathic Parkinson's disease: environmental and genetic evidence. Toxicol Sci.

[20] Nandipati S, Litvan I (2016). Environmental exposures and Parkinson's disease. Int J Environ Res Public Health.

[21] Ratner MH, Fitzgerald E (2017). Understanding of the role of manganese in parkinsonism and Parkinson disease. Neurology.

[22] Caudle WM (2017). Occupational metal exposure and parkinsonism. Adv Neurobiol.

[23] Goldman SM, Tanner CM, Olanow CW, Watts RL, Field RD, Langston JW (2005). Occupation and parkinsonism in three movement disorders clinics. Neurology.

[24] Fored CM, Fryzek JP, Brandt L (2006). Parkinson's disease and other basal ganglia or movement disorders in a large nationwide cohort of Swedish welders. Occup Environ Med.

[25] Fryzek JP, Hansen J, Cohen S (2005). A cohort study of Parkinson's disease and other neurodegenerative disorders in Danish welders. J Occup Environ Med.

[26] Kenborg L, Lassen CF, Hansen J, Olsen JH (2012). Parkinson's disease and other neurodegenerative disorders among welders: a Danish cohort study. Mov Disord.

[27] Mortimer JA, Borenstein AR, Nelson LM (2012). Associations of welding and manganese exposure with Parkinson disease: review and meta-analysis. Neurology.

[28] Racette BA, Tabbal SD, Jennings D, Good L, Perlmutter JS, Evanoff B (2005). Prevalence of parkinsonism and relationship to exposure in a large sample of Alabama welders. Neurology.

[29] Racette BA, Criswell SR, Lundin JI (2012). Increased risk of parkinsonism associated with welding exposure. Neurotoxicology.

[30] Andruska KM, Racette AB (2015). Neuromythology of manganism. Curr Epidemiol Rep.

[31] Samson K (2017). Manganese exposure, motor symptom progression may be dose-related. Neurology Today.

[32] Racette BA, Searles Nielsen S, Criswell SR, Sheppard L, Seixas N, Warden MN, Checkoway H (2017). Dose-dependent progression of parkinsonism in manganese-exposed welders. Neurology.

[33] Dlamini WW, Nelson G, Racette BA (2018). 1367 The association between parkinsonism and quality of life in South African manganese mine workers. Occup Environ Med.

[34] Rohani M, Kassiri N, Emamikhah Abarghouei M, Mohammadi S, Labbafinejad Y (2022). Prevalence of parkinsonism among foundry workers in an automobile manufacturing factory in Tehran. Cureus.

